# Population structure and distribution of geladas (*Theropithicus gelada*, Ruppell 1835) in Kotu forest, Northern Ethiopia

**DOI:** 10.1002/ece3.10206

**Published:** 2023-06-28

**Authors:** Degu Abate, Zerihun Girma

**Affiliations:** ^1^ Department of Natural Resource Management Mekdela Amba University Tuluawliya Ethiopia; ^2^ Wondo Genet College of Forestry and Natural Resource Hawassa University Wondo Gent Ethiopia

**Keywords:** age structure, band size, group size, habitat, sex ratio

## Abstract

Endemic gelada populations outside protected areas are less investigated, and population census data are not available. As a result, a study was conducted to investigate the population size, structure, and distribution of geladas in Kotu forest and associated grasslands, in northern Ethiopia. The study area was stratified into five dominant habitat types namely, grassland, wooded grassland, plantation forest, natural forest, and bushland based on dominant vegetation type. Each habitat type was further divided into blocks, and a total counting technique was used to count the individuals of gelada. The total mean population size of gelada in Kotu forest was 229 ± 6.11. The mean ratio of male to female was 1:1.178. The gelada age composition comprised is as follows: 113 (49.34%) adults, 77 (33.62%) sub‐adults, and 39 (17.03%) juveniles. The mean number of group one‐male unit ranged from 1.5 ± 0.2 in the plantation forest to 4.5 ± 0.7 in the grassland habitat. On the other hand, all‐male unit social system group was recorded only from grassland (1.5) and plantation forest (1) habitats. The average band size (number of individuals per band) was 45.0 ± 2.53. The largest number of geladas was recorded from grassland habitat 68 (29.87%), and the lowest was recorded from plantation forest habitat 34 (14.74%). Even though, the sex ratio was female biased, the proportion of juveniles to other age classes was very low compared with geladas in relatively well‐protected areas, indicating negative consequences for the future viability of the gelada populations in the area. Geladas were widely distributed over open grassland habitat. Therefore, for sustainable conservation of the geladas in the area, there is a need for integrated management of the area with special attention on the conservation of the grassland habitat.

## Graphical abstract

The total mean number of gelada in Kotu forest was 229 ± 6.11. The mean ratio of male to female was 1:1.178. Geladas were widely distributed over open grassland habitat.

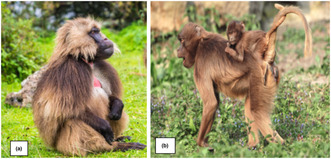

## 1 INTRODUCTION

**FIGURE 1:**
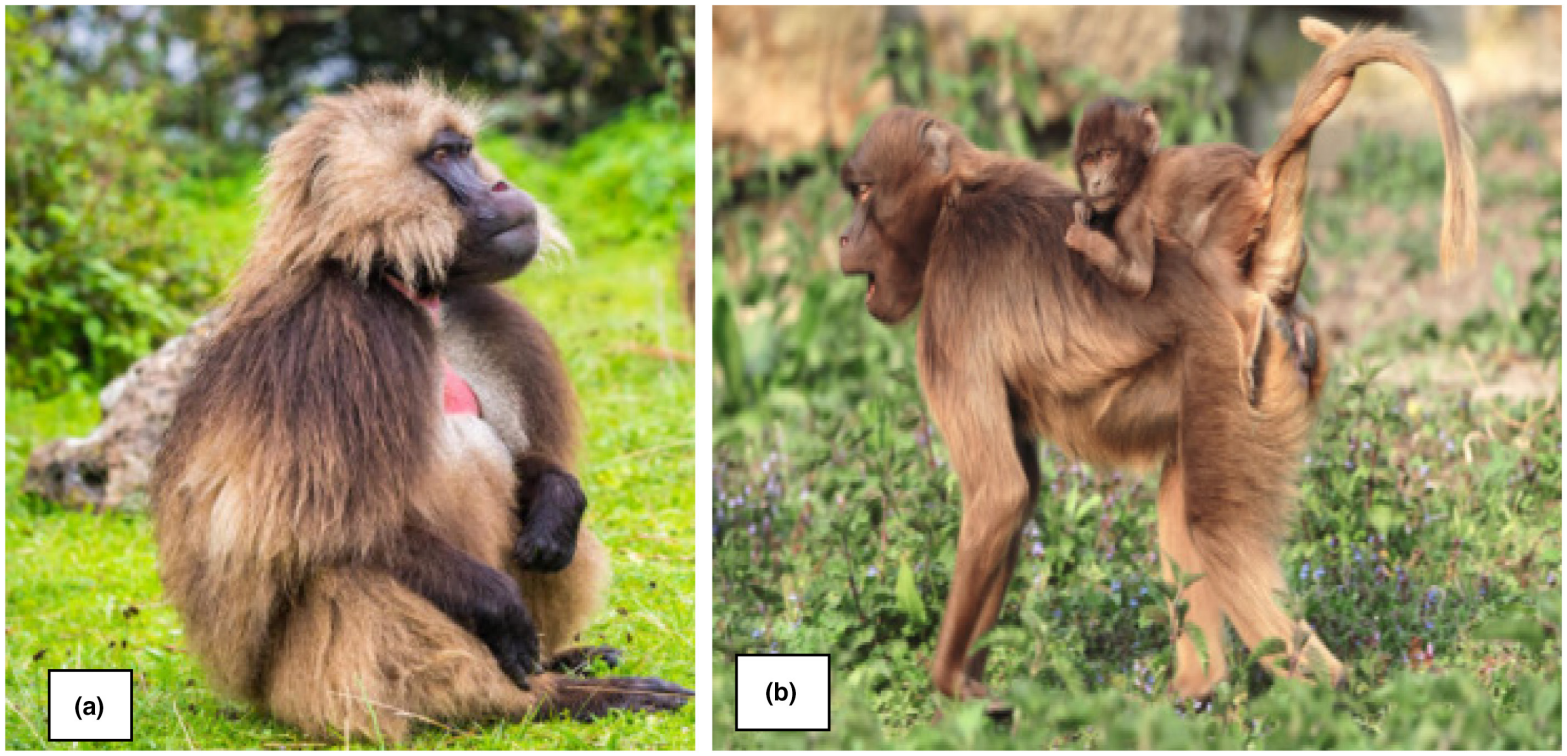
Adult male (a) and adult female with juvenile gelada (b).

Geladas are large primates with dark brown to buff coarse pelage, dark brown faces, and lighter pale eyelids (Ankel‐Simons, 2007). They exhibit marked sexual dimorphism, with adult females around two‐third the size of adult males (Figure 1). Gelada (*Theropithecus gelada*) is endemic to the northern and central plateau of Ethiopia, and its ancestors are once known to be widely distributed in Africa and Eurasia (Alba et al., 2014; Belmaker, 2010; Jolly, 1972). According to the analysis of mitochondrial DNA, geladas are known to closely relate to baboons (*Papio*), kipunjis (*Rungwecebus*), and crested mangabeys (*Lophocebus*; Zinner et al., 2018). The genus *Papio* and *Theropithecus* were believed to diverge about 4–5 million years ago from a common ancestor (Jolly, 1972).

There are two subspecies of geladas namely, *Theropithecus gelada gelada* and *Theropithecus gelada obscure*. The former occurs in the northern highlands, and the latter occurs in the central highlands of Ethiopia (Gippoliti, 2010; Yalden, 1983). The differences between *T.g. gelada* and *T.g. obscure* are minimal; there are a few visible differences. *T. g. obscure* has darker‐colored dorsal fur and flesh‐colored face (Belay & Shotake, 1998). This subspecies inhabits the north‐western area of the Great Rift Valley in Showa (Menz, Debrelebanos and Muger areas) and in the Wollo and Gojjam Provinces (Abie & Bekele, 2017; Moges, 2015; Yalden et al., 1996). However, *T.g. gelada* (Gippoliti, 2010) has a lighter fur appearance. This subspecies inhabits the northern highlands, particularly Simen Mountains National Park (SMNP) and associated highlands (Beehner et al., 2008; Ejigu & Bekele, 2014; Yalden et al., 1996; Yalden & Largen, 1992), and few populations have been recently reported from the highlands of Tigray (Girmay & Dati, 2020). According to Mori and Belay (1990) and Moges (2019), there is also a subpopulation of gelada distributed in the south‐eastern part of the rift valley in the Arsi Province near Bale Mountains National Park. Some morphological and genetic analyses showed that the Arsi gelada populations can be regarded as yet another distinct subspecies, *T. gelada arsi* (Belay & Mori, 2006; Gippoliti, 2010; Shotake et al., 2016; Zinner et al., 2018).

According to Mori and Belay (1990), the distribution of the gelada population on the Ethiopian plateau is associated with the availability of easily digested montane grasses. Geladas occur between an altitude of 1800 and 4400 m asl. The Blue Nile gorge and the upper Shebelle rift valley (east of the Bale massif) mark the western and south‐eastern borders and the range correspondingly (Gippoloti & Hunter, 2017). The largest population of gelada occurs in SMNP in Gonder Province (Asfaw & Subramanaian, 2013; Beehner et al., 2008; Woldegeoriges, 2015). The second largest population of gelada is found in Menz Guassa Community Conservation Area (MGCCA; Moges, 2015). More recently, populations of gelada are reported from Wof‐Washa Forest (Gosh‐Meda Area), central Ethiopia (Goshme & Yihune, 2018), from eastern escarpments of Tigray (Girmay & Dati, 2020), and Yegof Forest, South Wollo (Ahmed et al., 2022). Gelada population is limited to the high grassland cliff in the deep gorges in the central plateau, of the Wollo Province such as in Azwa and Arego valleys, Borena Sayint National Park (BSNP) and Mount Abune Yosef Community Conservation Area (MAYCSA; Adem, 2009; Ayalew, 2009; Eshete et al., 2015; Kifle, 2018). Population of gelada also occurs in the Wonchit valley between Showa and Wollo Provinces (Kifle et al., 2013). The southern isolated population of gelada is distributed over the valley and forest escarpments of Robe, Amigna, and Bele districts (woreda) in Arsi zone (Abu, 2011; Moges, 2019).

Geladas are one of the most multilevel societies found among primates (Snyder‐Mackler et al., 2012). They exhibit from simple primary‐level organization to the largest multilevel social structure. The primary level of organization comprises a one‐male unit (OMU) and an all‐male unit (AMU) (Snyder‐Mackler et al., 2010). The secondary‐level organization includes closely associated two or more OMUs and AMUs (Dunbar, 1984). The tertiary‐level organization encompasses multiple closely associated OMUs and AMUs (Dunbar & Dunbar, 1975). The multilevel organization is made of the association of any units that form bands (Snyder‐Mackler et al., 2012).

Geladas that live in protected areas received high conservation attention and have been studied very well at different times by different researchers (Beehner et al., 2008; Hunter, 2001; Woldegeoriges, 2015). However, populations of gelada have been known to face habitat degradation, fragmentation, and loss throughout their ranges (Abie & Bekele, 2017; Andarge, 2010; Moges, 2015; Yihune et al., 2009). Particularly, populations of gelada that live outside protected areas received little conservation attention (Kifle et al., 2013). On top of that, there is no sufficient study on populations of geladas that serves as a population stock source. There is a clear gap between the total population estimate (50,000–60,000 individuals, Beehner et al., 2008) and the actual population size, indicating the need to explore all possible ranges of the species to come up with the most reliable global population estimate.

Kotu forest is remnant dry evergreen Afromontane forest that comprised open grasslands, wooded grassland, bush lands and managed plantation forest. In the area anthropogenic activities such as livestock encroachments, logging and firewood collection caused geladas habitat degradation and fragmentation (Delanta Woreda Agricultural and Natural Resource Development Office (DWANRDO), 2016). Furthermore, there was an active mineral (Opal) extraction activity going on in the area that degraded the habitat of the gelada, especially the gelada's cliff (DWANRDO, 2016). This mineral naturally occurs embedded within rock in the cliffs and cliff edges (Rondeau et al., 2010), which are ideal habitat for gelada in providing cover against extreme temperatures, predator, serve as breeding sites, performing social activities and also used as cover during birthing time (Dunbar, 1978; DWANRDO, 2016). Geladas congregate along the cliff edges around sunrise (Beehner et al., 2008), and most gelada sexual activity occurs during the morning hours before midday (Dunbar, 1978). In the meantime, there is no exact population estimate and information on population distribution of geladas among dominant habitat types in the area.

In wildlife conservation, accurate population estimates of wild animal populations are essential for several reasons. Primarily, population estimates across a time will help to determine whether the population size of particular species in the given habitat is being maintained, in decline or recovery (Beehner et al., 2008). Secondly, establishing an accurate population count for each species is the first step in conservation and is important input for drafting effective wildlife policy. Thirdly, data on wildlife population estimate and description of its distribution are important input for managing human–wildlife conflict (Mokennen et al., 2010; Yihune et al., 2009), since most of the ranges of the species occur in human‐dominated landscapes. Therefore, the present study is aimed at determining the population size and population distribution of geladas among dominant habitat types of Kotu forest nexus for sustainable population conservation.

## 2 MATERIALS AND METHODS

### 2.1 Study area

**FIGURE 2:**
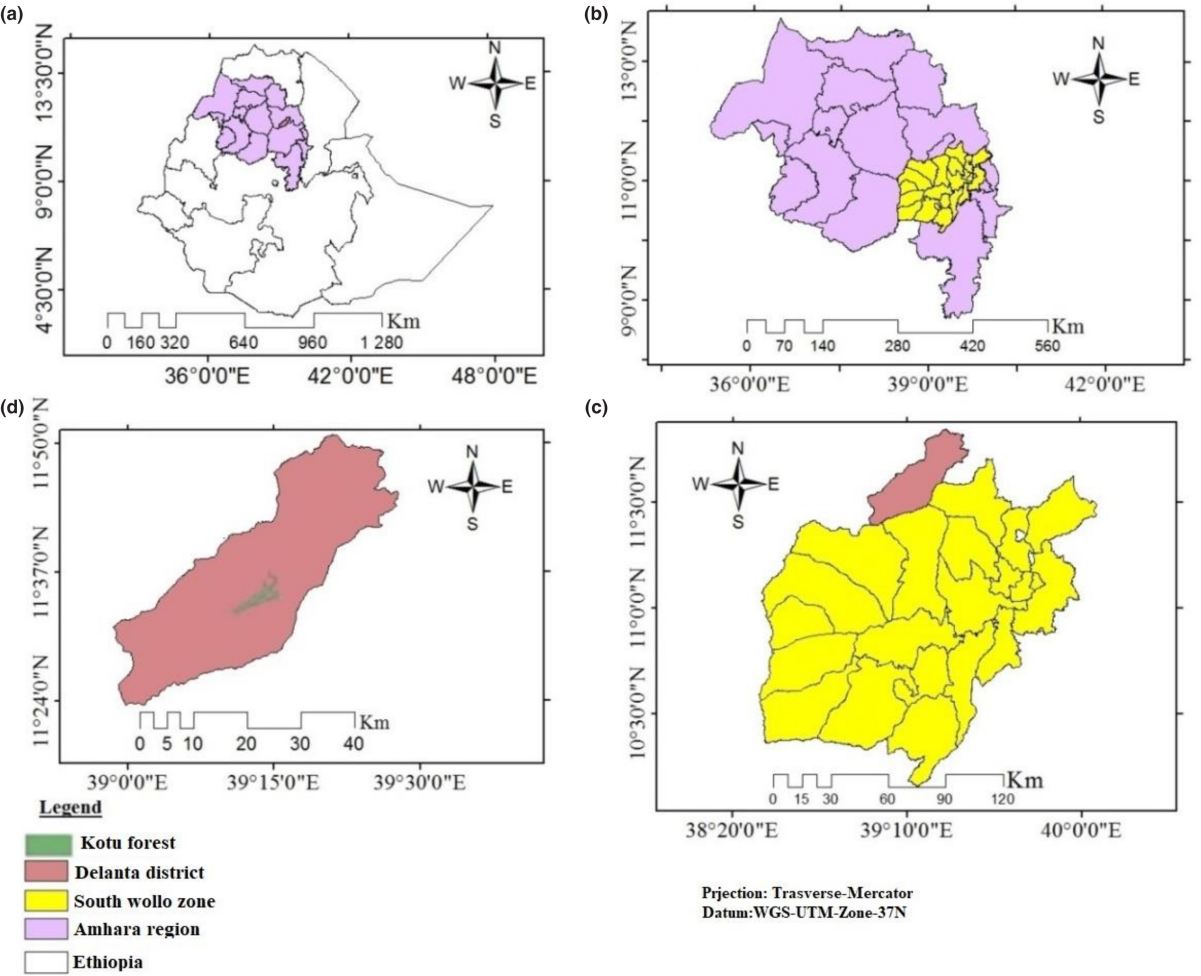
Location map of Ethiopia (a), Amhara region (b), South Wolo zone (district) (c), Delanta woreda and the study area (Kotu forest) (d).

Kotu forest is located in Delanta woreda (district), under South Wollo Zonal Administration of the Amhara National Regional State of Ethiopia (Figure 2). It is located at a distance of 499 km north of Addis Ababa. Kotu forest is geographically situated between 11°30′45″N to 11°35′45″N latitude and 39°11′50″E to 39°14′10″E longitude (Figure 2). The total area of the forest is 1374 ha with an altitude ranging from 2771 to 2987 m asl. The study area is geologically characterized by extensive escarpment plateaus, hills with small mountainous ridges, and gorges (DWANRDO, 2016). The mean monthly temperature of the study area is 13°C with a maximum temperature of 19.8°C and with a monthly mean minimum temperature of 6.8°C (Ethiopian National Meteorological Agency (ENMA), 2017). The study area is characterized by bimodal rainfall distribution patterns; with a long rainy season from June to October and a minor rainy season from March to May. It receives the highest rainfall (75%–80%) during the long rainy season (ENMA, 2017) and 20%–25% rainfall during the short rainy season. The area comprises five dominant habitat types namely; grassland, wooded grassland, natural forest (dry evergreen Afromontane forest), plantation forest, and bushland. *Acacia abysinica, Acacia salingna*, *Acacia decurrens*, *Olea europaea* and *Juniperous procera* are among the dominate tree species in the study area (DWANRDO, 2016).

#### 2.1.1 Grassland

This habitat is locally named “Ambanat.” This habitat is dominated by grass species. The grass species often consumed by the geladas such as *Cynodon aethiopicus*, *Andropogone abyssinicus*, *Pennisetum clandestinum*, *Digitaria abyssinica* predominantly occur in this habitat type. Its altitude ranges from 2900 to 2987 m asl. In this habitat type, there are permanent water sources and sufficient food resources better than in any other habitat type. However, illegal livestock grazing and grass cutting are widely practiced especially during the wet season (DWANRDO, 2016).

#### 2.1.2 Wooded grassland

This habitat type is dominated by grass species with scattered trees of *Acacia* spp. The dominant grass species in this habitat type that are consumed by geladas are *C. aethiopicus*, *P. clandestinum*, and *Sporobolus pyramidalis*. The habitat type is locally called “Minch” (local mean spring, due to its year‐round source of water). Its altitude is within the range of 2872–2901 m asl. In this site, harvesting of *Acacia* spp. for charcoal production was frequently observed (DWANRDO, 2016).

#### 2.1.3 Plantation forest

This habitat type is dominated by plantations of *J. procera* with intermixed grass and shrub species in understory vegetation. In this habitat type, grass species consumed by the geladas such as *A. abyssinicus* and *S. pyramidalis* were found sparsely. Its altitude ranges from 2902 to 2969 m asl. The habitat is locally called “Chichet.” In this habitat, human disturbance is intense due to the increased availability of logging trees such as *J. procera* and the availability of the mineral Opal (Rondeau et al., 2010). There was illegal harvesting of trees such as *J. procera* by the local communities for household consumption and for sale in the local market. The local community also constantly destructs the rocky cliff refugees of the gelada in search of the mineral Opal in the rock cracks (DWANRDO, 2016; Zhao & Bai, 2020).

#### 2.1.4 Natural forest (dry evergreen Afromontane forest)

This habitat type is situated within an altitudinal range of 2771 to 2810 m asl. It is locally named “Embisiareh” (locally means *Al. abyssinicus, Al. abyssinicus* is fairly abundant in this habitat type). Herb species consumed by the geladas such as *Trifolium temnense*, *Thymus schimperi* and *Plantago lanceolate* intermixed with scattered grass species such as *A. abyssinicus* sparsely covered the understory vegetation. The habitat is dominated by tree species such as *Acacia* spp, *O. europaea*, *J. procera* and *Al. abyssinicus* with some bushes and grasses forming the understory vegetation (DWANRDO, 2016).

#### 2.1.5 Bushland habitat

Bushland is locally named “Berewsirt.” Its altitude ranges from 2825 to 2891 m asl. This habitat type comprises deciduous and evergreen shrubs mainly *Dodonaea viscose* intermixed with grasses and other herbaceous vegetation forming the understory layer. Herb species consumed by the geladas such as *T. temnense* and *Plantago lanceolata* were rarely found (DWANRDO, 2016).

**FIGURE 3:**
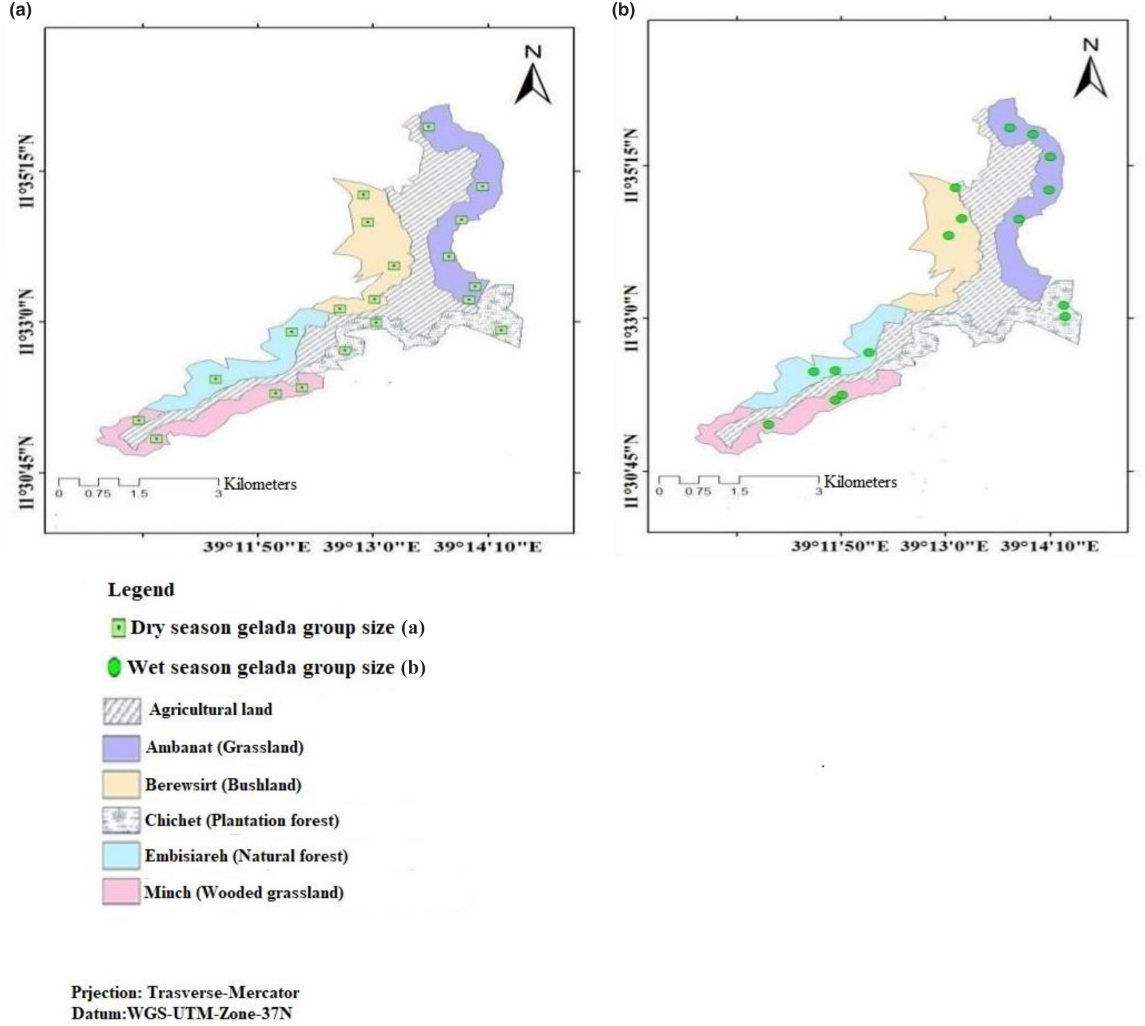
Distribution map of gelada groups among dominant habitat types in Kotu forest during Dry (a) and wet (b) seasons.

The Kotu forest comprises highly fragmented forest and associated grasslands surrounded by agricultural lands (Figure 3). The fragmented forest is exposed to multiple edge effects often highly influenced by the surrounding agricultural landscape. Early dry season (when the data collection was conducted) is crop‐harvesting time in the surrounding farmlands and geladas moved out of their cliffs for open grazing. As a result, the individuals of gelada were easily observed in the habitat. During the middle wet season (when the data collection was carried out), the surrounding agricultural lands were covered by crops and humans with their livestock moved to the forest introducing increased disturbances. Furthermore, during the wet season, the weather was cool and was often foggy or rainy, which promote the growth of abundant grasses in the various habitat types, including gelada cliff areas.

### 2.2 Sampling design and data collection

The study area was stratified into five dominant habitat types based on the dominant vegetation types in the area (Figure 3). The area coverage of each habitat type was as follows: grassland (336 ha, 24.45%), wooded grassland (226 ha, 16.45%), natural forest (dry evergreen Afromontane forest; 243 ha, 17.69%), plantation forest (251 ha, 18.27%), and bushland (318 ha, 23.14%). Proportional to the area coverage of each habitat type, each habitat was further subdivided into counting blocks. Grassland and bushland habitats were subdivided into six and five blocks, respectively, whereas plantation forest, natural forest, and wooded grassland were subdivided into four, four, and three blocks, respectively. Each habitat type was subdivided into counting blocks using natural boundaries and marks such as mountains, cliffs, springs, and valleys. Blocks were purposively established along cliff sides, where geladas sleep and, in a way, it catches the geladas as they come up off of their cliffs following Hunter (2001).

Data collection was carried out from August 2017 to February 2018 covering both dry and wet seasons; each block was visited (population counted) six times per season. The wet season data collection was conducted from August 2017 to October 2017, and the dry season data collection was carried out from December 2017 to February 2018.

The total count method of population census was used to determine the population size and structure of gelada in the study area. Total population census/count has been reported to be effective to determine the population size of gelada in open habitat type, where there is good visibility of individuals of gelada in various localities (Abu, 2011; Adem, 2009; Ayalew, 2009; Beehner et al., 2008; Girmay & Dati, 2020; Goshme & Yihune, 2018; Kifle et al., 2013; Moges, 2015). Likewise, the present study area is a predominately open habitat that allows reasonable visibility of individuals of gelada. More precisely, during morning hours, geladas aggregate along cliff edges for sun basking and feeding, where observers can easily see and count all individuals in a given area (Girmay & Dati, 2020). The total census was carried out in each habitat type on foot simultaneously from suitable vantage points or by moving along the habitat by involving well‐trained people in each habitat type to avoid double counting problems (Fashing & Cords, 2000; Mokennen et al., 2010). Since the simultaneous counting individuals' gelada overall habitat types and blocks demand the involvement of groups of people, people from the locality were purposively selected and trained. A total of 40 people were involved in the data collection. One‐day theoretical and 2‐day practical (on‐site) training were given to the trainees on the methodology of counting geladas and age and sex identification techniques following Beehner et al. (2008). All trainees received a secondary school education and had prior experience about the species sex and age identification and its behavior. A census was conducted only when gelada start moving to the cliff edge, and no individual remained on the cliff edges following Hunter (2001). All gelada population counting was conducted on the same day and at the same time during early morning (7:00–11:00 a.m.) hours following Beehner et al. (2008) & Mokennen et al. (2010). Even though, attempts were made to conduct counting during sunny and less foggy days to increase the visibility of geladas, foggy and rainy weather limited the visibility of individuals of gelada during the wet season.

Determination of age and sex structure was carried out according to the procedures of Beehner et al. (2008). The sex and age of individuals of gelada were identified based on body size and developmental characteristics of individual gelada. Identification was conducted using 7 × 40 Binoculars and with the naked eye through direct observation of individuals of gelada. The age and sex identifications were based on body size and morphological characteristics of the species (Beehner et al., 2008; Table 1).

**TABLE 1:** Peculiar characteristics of gelada used to distinguish different age‐sex classes (Beehner et al., 2008).

Age‐sex category	Characteristics
Adult female	The adult female is smaller in size than the adult male; it lacks cape and has more uniform pelage color than the male. It is characterized by hairless hourglass‐shaped red area of the skin located on the chest, which is surrounded by pearl‐like knobs of skin. The adult female skin of the neck and chest is pale and has paracallosal skin. The adult female is larger in size than the sub‐adult female
Adult male	The adult male is distinguished from others by its prominent manes. They are also about twice the body size of adult females and sub‐adult males
Sub‐adult male	The sub‐adult male is similar in size to adult females, but with initial development of manes and cape hair light in color extending just past the shoulders. In sub‐adult male the cheek tufts are present, but not extending below the chin. Their ears are highly visible and their surrounding fur around the chest is patch gray‐brown in color
Sub‐adult female	The sub‐adult female is smaller in size than the adult female and sub‐adult male. The sub‐adult female has light pelage black skin
Juveniles	Juveniles are immature individuals from 0.5 to 3.5 years of age often closely associated with adult females for parental care and have darker pelage color. If the age‐sex class of geladas were out of the above four categories, they were categorized as juveniles

During the population census, the group size of gelada groups was recorded before treating the individuals in the groups into respective age and sex categories. Identified individuals were recorded on a separate data sheet as adult males, adult females, sub‐adult males, sub‐adult females, and juvenile (young). Geladas exhibit extremely multilevel social organization. Gelada society is an extremely flexible, multilevel society with fission–fusion dynamics, and as such gelada society presents an unusual example for understanding the evolution of modular societies (Snyder‐Mackler et al., 2012). A group of gelada is a multilevel social order that consists of individuals of gelada usually composed of a leader male, several adult females, and their offspring (Gippoloti & Hunter, 2017). Those individuals seen within a distance of <50 m from the nearby group were recorded as members of the same group. To determine group size and composition once a group was spotted, it was categorized based on social organization and grouped into one of the following units: OMU and AMU. OMU group is defined as a group of gelada that consists of a leader male, several adult females, and their offspring in a specific location, whereas AMU group is defined as groups containing only bachelor sub‐adult males and old adult males (2–15 males) and led by a single male individual and normally contains one young adult and several sub‐adult males (Gippoloti & Hunter, 2017). A band of gelada is defined as an association of two or more adult males with their followers (individuals of gelada such as adult and sub‐adult females, sub‐adult males and young) that live together in their home range (Beehner et al., 2008). Bands are not discrete entities of units that are found together every day; they come together occasionally for grazing opportunities and social grooming. The band is made up of multiple reproductive units and all‐male groups (Dunbar & Dunbar, 1975).

To determine the relative distribution pattern of gelada, an intensive ground inspection was conducted in each habitat type and in each block during the continuous daily counting periods following Groves (2005). The GPS location (coordinate points), group size, altitude, and habitat type were recorded. During the survey, 6–8 coordinates points of all non‐overlapping gelada group ranges (distinct range exclusively occupied by a certain group) were recorded during both wet and dry seasons.

### 2.3 Data analysis

Statistical Package for Social Science (SPSS) version 16 computer software program was used for all statistical analysis. All statistical tests were two‐tailed with 95% of confidence intervals, and the rejection level was *p* ≤ .05. Mann–Whitney *U*‐test was used to test the population count among wet and dry seasons. Chi‐square test was used to test if there is a significant variation between the number of males and females. It was also used to test if there is a significant variation in the number of individuals of gelada among habitat types. During the total count identifying the group of gelada and counting the number of individuals that form the group was a base for estimating the population density of gelada in the study area (Bocian, 1997).

(1) $$\text{Population density}=\frac{\text{Number of individuals that were counted in the area}}{\text{Total area}}$$

The population density of geladas in each habitat type in both wet and dry seasons was calculated and expressed as the number of individuals per km^2^.

Non overlapping ranges of groups of geladas were mapped using *x* and *y* coordinates collected using GPS and were mapped using Arc GIS software. In addition, the population count at each dominant habitat type was used to supplement the distribution pattern of gelada mapping. To determine the distribution pattern of wild animal populations, different researchers used the relative frequencies of observation of the animal in each habitat type (Abie & Bekele, 2017; Goshme & Yihune, 2018; Yazezew et al., 2011).

## 3 RESULTS

### 3.1 Population size and density

The total mean number of geladas in Kotu forest was 229 ± 6.11. They were 259 ± 6.77 and 198 ± 5.44 individuals of gelada during the dry and wet seasons, respectively. There was a significant variation in population size between wet and dry seasons (*U* = 1330.0, *p* = .001). The mean population density of gelada in the study area was 16.63 ± 2.21 individuals per km^2^ (Table 2). The population density of gelada was 14.41 ± 2.1 and 18.85 ± 2.4 per km^2^ during the wet and dry seasons, respectively.

**TABLE 2:** The population density of individuals of gelada per km^2^ in each habitat type during wet and dry seasons at Kotu forest (Mean **±** SE).

Local names	Habitat type	Population density per seasons (mean ± SE)		
		Wet	Dry	Mean
Ambanat	Grassland	4.38 ± 0.36	5.53 ± 0.24	4.96 ± 0.56
Minch	Wooded grassland	2.88 ± 0.21	3.78 ± 0.32	3.33 ± 0.45
Chichet	Plantation forest	2.16 ± 0.16	2.74 ± 0.11	2.45 ± 0.29
Embisiareh	Natural forest	2.33 ± 0.21	2.91 ± 0.15	2.62 ± 0.29
Berewsirt	Bushland	2.66 ± 0.24	3.89 ± 0.38	3.27 ± 0.62
Total		14.41 ± 2.1	18.85 ± 2.4	16.63 ± 2.21

### 3.2 Age and sex structure

**FIGURE 4:**
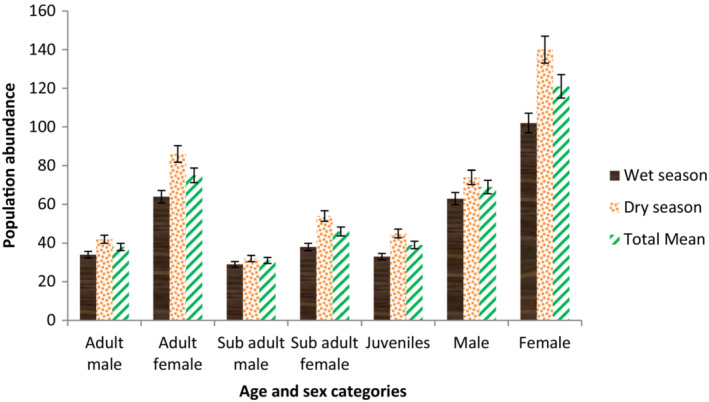
Age and sex structure of gelada population during wet and dry seasons (Mean ± SE) in Kotu forest.

Out of the total mean number of 229 ± 6.11 individuals of the gelada encountered in the study area, females comprised 121 ± 18.8 (52.84%) individuals and males 69 ± 5.0 (30.13%) individuals, whereas 39 ± 5.85 (20.52%) individuals were juveniles with undetermined sex (Figure 4). The number of females was significantly higher than males (*χ* ^2^ = 14.23, df = 1, *p* < .001). There was a larger number of female individuals during the dry (140 ± 16.10) season than the wet season (102.0 ± 13.10). Out of the total mean individuals of gelada recorded in the study area, adult, sub‐adult, and juveniles constituted 113 (49.34%), 77 (33.62%), and 39 (17.03%), respectively. The adult:sub‐adult:juvenile ratio is 2.8:1.9:1.

The population was dominated by adult females (75 ± 10.90), while sub‐adult males (31 ± 1.75) were least represented (Figure 4). Adult females comprised the largest proportion of geladas during both dry (86 ± 2.67, 33.20%) and wet (64 ± 1.88, 32.32%) seasons (Figure 4). On the other hand, sub‐adult males comprised the least proportion of geladas during both dry (32 ± 1.02, 12.36%) and wet (29 ± 0.86, 14.64%) seasons (Figure 4). The mean male‐to‐female sex ratio was 1:1.78. The average ratio of the adult males to adult females was 1:1.96 (Table 3).

**TABLE 3:** Age and sex ratio of gelada during the wet and dry seasons at Kotu forest.

Seasons	Age and sex ratio					
	AM: AF	SAM:SAF	SAM:AM	SAF:AF	JUV:AF	M:F
Wet	1:1.88	1:1.31	1:1.17	1:1.69	1:1.94	1:1.64
Dry	1:2.04	1:1.69	1:1.24	1:1.59	1:1.91	1:1.89
Mean	1:1.96	1:1.50	1:1.21	1:1.64	1:1.91	1:1.78

Abbreviations: AF, adult females; AM, adult males; F, females; JUV, juveniles; M, males; SAF, sub‐adult females; SAM, sub‐adult males.

### 3.3 Group numbers/population structure and composition

The mean number of group OMU ranged from 1.5 ± 0.2 in the plantation forest to 4.5 ± 0.7 in the grassland habitat. On the other hand, AMU social system group was recorded only from grassland (1.5) and plantation forest (1) habitats (Table 4). During the wet season, 16 groups were encountered (Figure 3); out of this, 2 (12.5%) were AMU (bachelor and sub‐adult male), and 14 (87.5%) were OMU social system. During the dry season, 20 groups were encountered (Figure 3), out of which 3 (15%) were AMU and 17 (85%) were OMU social system. The average maximum AMU group number was 11.5 ± 0.5, and the average minimum was 3.5 ± 0.5.

**TABLE 4:** Mean OMU and AMU number of groups and composition of gelada at Kotu forest from August 2017 to February 2018.

Habitat type	OMU (AMU)							
	Mean number of groups	AF	AM	SAF	BM	OAM	Juveniles	Total
Grassland	4.5 *±* 0.70 (1.5 ± 0.25)	30.5 ± 1.60	13 ± 0.35	19 ± 0.83	4.5 ± 0.11	1.5 ± 0.06	14 ± 0.63	82.5 ± 3.21 (16.5 ± 3.25)
Bushland	3.5 *±* 0.50	19.5 ± 1.10	9 ± 0.57	9.5 ± 0.57	3.5 ± 0.01	0.5 ± 0.05	10.5 ± 0.52	52.5 ± 2.82
Plantation forest	1.5 *±* 0.20 (1 ± 0.16)	9.5 ± 0.98	5 ± 0.25	6.5 ± 0.49	5.5 ± 0.10	1 ± 0.28	4.5 ± 0.41	32 ± 2.51 (10 ± 1.25)
Natural forest	2 *±* 0.30	7.5 ± 0.50	4 ± 0.12	4.5 ± 0.49	6.5 ± 0.25	1.5 ± 0.06	3 ± 0.20	29 ± 1.62
Wooded grassland	2 *±* 0.30	8 ± 0.70	7 ± 0.46	4.5 ± 0.47	6 ± 0.13	0.5 ± 0.05	7 ± 0.44	33 ± 2.25
Overall mean	13.5 ± 1.5 (2.5 ± 0.5)	75 ± 4.9	38 ± 1.75	46 ± 2.85	26 ± 0.6	5 ± 0.5	39 ± 2.2	202 ± 12.5 (26.5 ± 4.5)

*Note*: The number in brackets shows AMU (all‐male unit). AF, AM, SAF, BM, OAM, juveniles, and total are mean values per habitat type.

Abbreviations: AF, adult female; AM, adult male; BM, bachelor male; OAM, old adult male; OMU, one‐male unit; SAF, sub‐adult female.

The average band size was 45.0 ± 2.53. The band size during the wet season was 2–4 leader males with up to 60 individual followers. On the other hand, during the dry season, the band size and composition comprised most of the time 2–8 leader males with up to 110 individual followers.

### 3.4 Population distribution pattern

**FIGURE 5:**
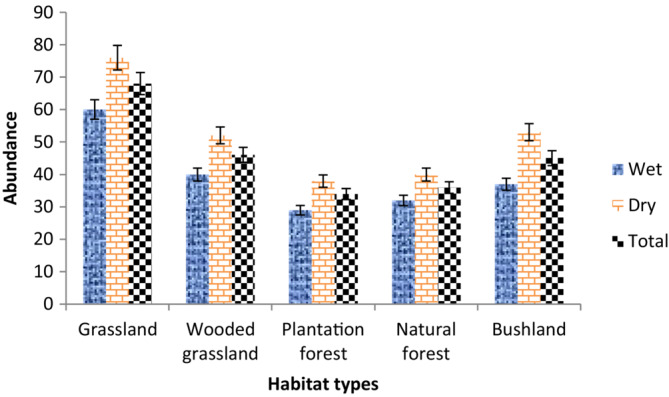
Population abundance of geladas among different habitat types during wet and dry seasons (Mean ± SE) in Kotu forest.

Populations of gelada were not uniformly distributed over the study area among dominant habitat types. The largest (68 ± 8.0, 29.87%) number of gelada was observed in the grassland habitat, while the least (34 ± 4.5, 14.74%) were recorded in the plantation forest habitat (Figure 5). Similarly, the largest (4.96 ± 0.56) density of gelada was recorded in grassland habitat, while the least (2.45 ± 0.29) was in plantation forest (Table 2). There was a significant difference in the number of geladas among habitat types (*χ* ^2^ = 15.913, df = 4, *p* = .003). During the wet season, 16 groups of geladas comprising 198 individuals were recorded in Kotu forest (Figure 3). Out of these, the largest (5, 31.25%) group was recorded from the grassland habitat and the lowest (2, 12.50%) group in the plantation forest (Figure 3). During the dry season, 20 groups comprising 259 individuals were recorded. During the dry season, the maximum (6, 30.0%) number of group was recorded from grassland habitat, and the minimum (2, 10.0%) number of group was recorded from the natural forest (Figure 3).

## 4 DISCUSSION

The study has revealed that Kotu forest supports a viable population of geladas, although isolated from other subpopulations in the nearby fragmented habitats of gelada. However, the area supports fewer subpopulation of gelada (mean population size 229) compared with the population in other ranges of gelada (Beehner et al., 2008, 4620 individuals in SMNP; Adem, 2009, 529 individuals in BSNP; Ayalew, 2009, 914 individuals in Azewa and Harego valleys; Abu, 2011, 338 individuals in Arsi; Moges, 2015, 1502 individuals in MGCCA, Abie & Bekele, 2017, 1608 individuals in Debrelibanos and Goshme & Yihune, 2018, and 435 individuals in Wof‐Washa Forest). This could be attributed to the current study area's smaller size in comparison with the species' other ranges, and the habitat is characterized by ravine topography, which opens into farmlands in all directions. The species' habitat in the current study area is also drier and less productive than most other habitats found throughout the species' range. Furthermore, threats such as habitat destruction in search of the mineral opal and deforestation, retaliatory killings against crop damage, which are intense in the present area (DWANRDO, 2016) compared with its other ranges, could reduce the population size. However, the population size is larger than the recent report from eastern escarpments of Tigray (Hawzien and Ganta‐Afeshum disricts), which is 105 individuals (Girmay & Dati, 2020). This could be because the species habitat in Tigray (northern Ethiopia) is drier and with poor habitat quality.

A significantly higher population of gelada was recorded during the dry season than the wet season unlike the populations of the species over some other ranges. For example, there were no significant seasonal variations in populations of gelada in Gich, SMNP (Woldegeoriges, 2015), Debrelibanos, central Ethiopia (Abie & Bekele, 2017), Wof washa, central Ethiopia (Goshme & Yihune, 2018). However, in line with the present study some studies carried out on population surveys of geladas in various localities such as Wochit valley (Kifle et al., 2013), eastern escarpments of Tigray (Girmay & Dati, 2020), and Yegof National Forest Priority Area (Ahmed et al., 2022) reported significant variations in the population of geladas among wet and dry seasons. The seasonal variation is mainly attributed to the reproductive and crop‐raiding behavior of the species and the weather condition in the particular study area.

Normally, geladas give birth after the wet season during the early dry season (Ejigu & Bekele, 2014; Roberts et al., 2017). More categorically, both ecological and social birth peak operates in geladas. According to a study carried out by Tinsley et al. (2018) on gelada reproductive behavior, about 37.4% of all ecological births (non‐takeover births) occurred between August and October, peaking in September and October. Ecologically birth peak is more closely related to peak food availability. It was also reported that social (takeover) births peaked between December and February (54.4%; Tinsley et al., 2018). Social birth peak synchronized with the dry season data collection period, which could increase the number of young ultimately increasing the total population during the dry season.

The most plausible explanation comes from the fact that there is intense agricultural encroachment surrounding the habitats of geladas in the present study area. Because the wet season is a crop‐growing season, geladas were observed to widely involve in crop‐raiding behavior during the wet season. Gelada is identified as one of crop raider primates over most of its ranges (Moges, 2015; Yihune et al., 2009). The diet of geladas is predominantly composed of grasses and rhizomes (Moges, 2015). However, cereal crops may be taken as an alternative food source where agriculture encroaches onto the habitat of geladas (Yihune et al., 2009). Farmers use guard animals like dogs and stone at individuals of gelada to chase them away from their farmlands during the wet season to prevent crop damage. This practice may result in fewer sightings of the individuals of gelada during the wet season. During the dry season, crops are harvested and this enables geladas to roam freely among all habitat types. Similarly, Kifle et al. (2013) recorded fewer individuals of geladas during the wet season than the dry season in Wonchit valley and discussed that this could be attributed to the fact that the wet season is a cultivation season and hence, farmers have to chase away geladas far away from cropland and most of their open habitat types. Likewise, a study at Yegof National Forest Priority Area (Ahmed et al., 2022) also reported less number of gelada counted during the wet season than the dry season and attributed the variation due to intense agricultural activity in the area during wet season that leads to chasing of geladas.

Furthermore, the foggy weather conditions during the wet season compared with a bright sunny day during the dry season could reduce the sighting of the animals during the wet season (Ahmed et al., 2022). The study area receives 75–80% rainfall during the wet season (ENMA, 2017). This high amount of rain and fog could affect the probability of detecting individuals of gelada in the study area. According to Hunter (2001), excessive rainfall causes stress among the gelada populations, to overcome this heavy rainfall geladas aggregate themselves in their cliff.

Understanding sex and age population structure is important for evaluating the viability of the species as these variables reflect the structure and the dynamics of a species population (Groves, 2005). The male‐to‐female gelada sex ratio in the study area revealed female‐biased ratio (the adult male‐to‐female ratio is 1:1.96) and unequal mean ratio of juveniles to other age classes. The result is consistent with other studies (e.g., Woldegeoriges 2015) (the adult male‐to‐female ratio is 1:3) at Gich area of the Simen Mountain National Park, Abie & Bekele, 2017 (the adult male‐to‐female ratio is 1:3.57) at Deberelibanos highlands, central Ethiopia and Moges, 2019 (the adult male‐to‐female ratio is 1:4.1 in Arsi). The female‐biased ratio is a characteristic of a naturally growing population under normal circumstances (Beehner et al., 2008). Secondly, the reduced number of male geladas could be attributed to retaliatory killings induced by the species involvement in intense crop‐raiding behavior; this was observed during the data collection period, particularly during wet season. Geladas primarily eat leaves and grass, although they opportunistically eat cereal crops where agriculture abuts their habitat (Kifle et al., 2013; Moges, 2015). In the study area male gelada primarily bachelor males are hold and regularly try to snatch crops from nearby agricultural farmland traveling far distances and separating themselves from the group. The farmers use wire traps and kill them (personal observation, 2017). This could be the main cause for the reduced number of adult males compared with adult females gelada in the study area. Likewise, a study conducted in Wonchit valley showed male geladas to engage more in crop‐raiding activities due to this behavior the local farmer used a snare to kill them, due to this the number of males in the area was small compared with the number of adult females (Kifle, 2018). Furthermore, the natural predator caracal, common jackal, spotted hyena, leopard, and serval cat have been observed to be frequent over most of the habitats of gelada (researchers observation during data collection). During data collection period attempts to prey on adult and sub‐adult males and young by these predators were encountered. In line with this, Roberts and Dunbar (1991) reported that the male, especially bachelor and sub‐adult male geladas are always vulnerable to predators. A recent study in Guassa Community Conservation Area indicated a significant predation risk of geladas by a leopard (Lin et al., 2020). However, the reduced number of females and young compared with the above studies carried out elsewhere over the ranges of geladas could indicate reduced population growth and signal immediate conservation attention.

Unlike the present study, studies among relatively better‐protected ranges of geladas such as Simen Mountains National Park (Beehner et al., 2008), Guassa Community Conservation Area (Moges, 2015) Debrelibanos, central Ethiopia (Abie & Bekele, 2017) revealed more proportion of young and infants than adults. The possible reason for the small proportion of juveniles in the study area could be less availability of food resources (due to immense anthropogenic activities) that could limit the reproduction success. Dunbar (1978) discussed that food availability determines the birth rate of gelada population. Furthermore, unproductive female geladas may exist in the population that could reduce the population of juveniles. Dunbar (2002) revealed that the existence of unproductive female gelada attributes to the existence of a small number of juveniles in gelada populations.

The mean group number of gelada (16) encountered at Kotu forest was higher than Chenek, Simien Mountains National Park (10.5) (Ejigu & Bekele, 2014) and Wof Washa Forest (Goshme & Yihune, 2018; 10.4). However, fewer mean group size of gelada was encountered than Wonchit valley (23.01; Kifle et al., 2013). According to Ohsawa and Dunbar (1984) the number of individuals in a group of gelada is mainly dependent on the social factors that are related to group congregation. Variations in group size are the results of the cost and benefits of group congregation mainly associated with food and predator availability (Ejigu & Bekele, 2014; Majolo et al., 2008). Generally, larger groups benefit from defending predators and smaller groups benefit from less competition for food. The relatively higher number of groups of gelada encountered in the present study area is probably attributed to the risk of predation from carnivores such as caracal, common jackal and spotted hyena that were observed to frequently prey on geladas during the study period (Researcher observation). On the other hand, the higher number of groups of gelada and band size during the dry season than the wet season could be due to less availability of palatable grasses during dry season than wet season.

The distribution pattern of geladas in the study area was not uniform and varied depending on the food and water availability. The largest number of gelada was recorded in the open grassland habitat, while the least was in the plantation forest habitat. Gelada distribution is based on food availability, quality, and distance from the human settlement (Wallace, 2006). According to Goshme and Yihune (2018) and Ahmed et al. (2022) studies, open grassland is the preferred habitat for geladas. It is well known that grass species make up the largest portion of geladas diet (Eshete et al., 2015; Moges, 2019; Woldegeoriges, 2015). Grass species such as *C. aethiopicus*, *A. abyssinicus*, and *P. clandestinum* are highly preferred forage species by the geladas (Moges, 2015; Puff & Nemomissa, 2015) and are commonly abundant in the grassland habitat but incredibly rarer in plantation forest habitat.

It is a well‐established fact that grasses and rhizomes comprise the predominant diet of geladas (e.g., Dunbar, 1978, 1992; Fashing et al., 2014; Moges, 2015). Furthermore, the grassland habitat also offers a permanent source of water for the geladas. The result is in line with other studies such as Dunbar (1992), Abie and Bekele (2017) and Goshme and Yihune (2018). Furthermore, the grassland habitat preference could be related to the open nature of the habitat that increases vigilance. The open nature of the habitat enables gelada to take a measure to avoid predators through visual communication. Studies over different ranges of geladas concluded that open grassland is the most preferred habitat of geladas (Ahmed et al., 2022; Ejigu & Bekele, 2014).

Geladas in Kotu forest and associated grasslands are isolated populations where there is no direct dispersal opportunity to neighboring populations. There are intense human disturbances such as firewood collection, agricultural expansion, opal mining, grass cutting, and livestock encroachments that fragmented the habitats of geladas over years (Rondeau et al., 2010). These threats are very frequent in most highlands of Ethiopia (Girma et al., 2012; Hassain et al., 2008). Two main microevolutionary processes influence genetic patterns in declining and fragmented populations: genetic drift and gene flow. Genetic drift causes random fluctuations of allelic frequencies and loss of genetic diversity through time as a function of effective population size; dispersal‐mediated gene flow can buffer these effects in local populations. The isolated population in the present study area with no opportunities for dispersal‐mediated gene flow could lead to demographic and genetic stochasticity over decades. This could probably lead to local extinction in the worst scenario.

## 5 CONCLUSION AND RECOMMENDATIONS

The study revealed the presence of an isolated population of endemic gelada at Kotu forest and associated grasslands. The population sex ratio was female‐biased, indicating the potential for population growth, through adding new individuals into the population. However, the proportion of juveniles to other age classes was low compared with other localities of gelada population studies. Such a small number of juveniles will have negative consequences for the future viability of the gelada population in the study area. Geladas distribution is highly governed by habitats components such as water, food cover, and presence of threats. As a result, open grassland habitat is the most preferable habitat in the study area.

For sustainable conservation of the geladas in the study area, there is a need for integrated management of the area with special attention to the conservation of the grassland habitats and rock cliffs. Disturbances such as livestock grazing, firewood collection and opal extraction should be halted if possible or managed well. There is also a need for declaring the area as a protected area interconnecting with other ranges of geladas in the surroundings.

## AUTHOR CONTRIBUTIONS

**Degu Abate:** Data curation (lead); investigation (equal); methodology (supporting); writing – original draft (supporting). **Zerihun Girma:** Conceptualization (lead); formal analysis (equal); investigation (equal); methodology (equal); supervision (lead); writing – original draft (equal); writing – review and editing (lead).

## FUNDING INFORMATION

The research was conducted with finacial support from Ethiopian Ministry of Education.

## CONFLICT OF INTEREST STATEMENT

We the authors declare no competing interests.

## Data Availability

The data that support the findings of this study is archived Zenodo data repository by the file name Gelada Ecol. and Evol. With DOI: 10.5281/zenodo.8063755.
